# Structural insights into the gating mechanism of human SLC26A9 mediated by its C-terminal sequence

**DOI:** 10.1038/s41421-020-00193-7

**Published:** 2020-08-10

**Authors:** Ximin Chi, Xueqin Jin, Yun Chen, Xiaoli Lu, Xinyu Tu, Xiaorong Li, Yuanyuan Zhang, Jianlin Lei, Jing Huang, Zhuo Huang, Qiang Zhou, Xiaojing Pan

**Affiliations:** 1 Key Laboratory of Structural Biology of Zhejiang Province, Institute of Biology, Westlake Institute for Advanced Study, School of Life Sciences, Westlake University, 18 Shilongshan Road, Hangzhou, Zhejiang 310024 China; 2 State Key Laboratory of Membrane Biology, Beijing Advanced Innovation Center for Structural Biology, Tsinghua-Peking Joint Center for Life Sciences, School of Life Sciences, Tsinghua University, Beijing, 100084 China; 3 State Key Laboratory of Natural and Biomimetic Drugs, Department of Molecular and Cellular Pharmacology, School of Pharmaceutical Sciences, Peking University, Beijing, 100191 China; 4 Technology Center for Protein Sciences, Ministry of Education Key Laboratory of Protein Sciences, School of Life Sciences, Tsinghua University, Beijing, 100084 China; 5 Key Laboratory for Neuroscience, Ministry of Education/National Health and Family Planning Commission, Peking University, Beijing, 100191 China

**Keywords:** Molecular biology, Cryoelectron microscopy

## Abstract

The human SLC26 transporter family exhibits various transport characteristics, and family member SLC26A9 performs multiple roles, including acting as Cl^–^/HCO_3_^–^ exchangers, Cl^–^ channels, and Na^+^ transporters. Some mutations of SLC26A9 are correlated with abnormalities in respiration and digestion systems. As a potential target colocalizing with CFTR in cystic fibrosis patients, SLC26A9 is of great value in drug development. Here, we present a cryo-EM structure of the human SLC26A9 dimer at 2.6 Å resolution. A segment at the C-terminal end is bound to the entry of the intracellular vestibule of the putative transport pathway, which has been proven by electrophysiological experiments to be a gating modulator. Multiple chloride and sodium ions are resolved in the high-resolution structure, identifying novel ion-binding pockets for the first time. Together, our structure takes important steps in elucidating the structural features and regulatory mechanism of SLC26A9, with potential significance in the treatment of cystic fibrosis.

## Introduction

Solute carrier family 26 proteins function as anion transporters and/or channels^1,2^. Malfunction of SLC26 proteins is correlated with various diseases, such as congenital chloride diarrhea^3^ and deafness^4–6^. SLC26A9 is widely expressed in the respiration pathway^7^, digestion system^8,9^, kidney^10^, and neuron system^11^. With a natural location near cystic fibrosis transmembrane conductance regulator (CFTR) and functional similarity, SLC26A9 is believed to be a potential substitution for abnormal CFTR in cystic fibrosis^12,13^. There is a debate about whether SLC26A9 is a channel or transporter. It has been shown to be a constitutively active chloride channel that maintains chloride secretion in both epithelial cell lines^14^ and transiently transfected HEK293F cells^12^. In addition to chloride channel activity, SLC26A9 is also reported to behave as a Cl^–^/HCO_3_^–^ exchanger^15,16^ or sodium transporter^11^. In the stomach, exchanger activity is fundamental for gastric lumen pH regulation^17^. The expression level of SLC26A9 is increased upon *Helicobacter pylori* infection, indicating the role of mucosa protection^17^. The potential inhibition of SLC26A9-induced HCO_3_^–^ secretion by NH_4_^+^ further demonstrates the possible side effects of *Helicobacter pylori* infection^16^. Single nucleotide polymorphisms of SLC26A9 have different effects on its transport activity or subcellular localization, which also leads to its relevance to pathophysiology^18^. In addition, two diffuse idiopathic bronchiectasis-related missense mutations (R575W and V486I) were reported to decrease Cl^–^ transport, suggesting a potential role for SLC26A9 in treating cystic fibrosis-like diseases^19^. In SLC26A9-depleted animals, the excretion of Cl^–^ is impaired. Hypertension is observed under baseline conditions, which illustrates the possible role of SLC26A9 in salt homeostatic regulation^10^.

The transport activity of SLC26A9 is regulated in numerous ways. Direct physiological interactions with CFTR have been reported^20,21^, although there are conflicts about the regulatory effects of SLC26A9 on CFTR^12,22,23^. Research has also pointed out that the exact effect may be dependent on cell polarity and background. While in polarized cells, the interaction with CFTR seems to promote Cl^–^ secretion, Cl^–^ conductance is inhibited by CFTR activation in nonpolarized cell lines^22^. Calmodulin is proposed to regulate SLC26 family proteins through binding to intrinsically disordered regions^24^, which is consistent with a report of intracellular calcium inhibition of SLC26A9-associated currents^25^. In addition to protein–protein interactions, posttranslational modification also engages in regulation. Phosphorylation of SLC26A9 by WNK kinases can significantly decrease its plasma membrane localization and hamper its channel activity^26^.

The crystal structure of SLC26Dg, a bacterial homolog of SLC26, was reported in 2015, its transmembrane (TM) domain adopts a fold similar to that of UraA and NBCe1^27^. The cryo-EM structures of mouse Slc26a9 were reported recently^28^, revealing its dimerization mechanism. However, the C-terminal sequence was truncated in the mouse Slc26a9 protein to improve the protein stability and was therefore not visible in the structure. Here, we solved a cryo-EM structure of full-length human SLC26A9 at an overall resolution of 2.6 Å. The higher resolution of the structure provides more detailed information about the dimerization. Moreover, the C-terminal sequence of SLC26A9 is bound in the cytosolic entry of the putative ion-conducting pathway, implying its regulatory role in the protein. The electrophysiological characteristics of the C-terminus in the gating of SL26A9 indicate that the C-terminus can inhibit ion conduction. Therefore, SLC26A9 has a self-inhibition mechanism. Several nonprotein densities are resolved as bound ion or water molecules in the structure. With molecular dynamics simulation results, novel sodium and chloride ions were identified near the traditional substrate-binding pocket and extracellular side. Together, our structure and functional analysis reveal a new regulatory mechanism of SLC26 family proteins involving the C-terminal sequence and unreported ion-binding pockets.

## Results

### Overall structure and domain assembly of human SLC26A9

**Fig. 1:**
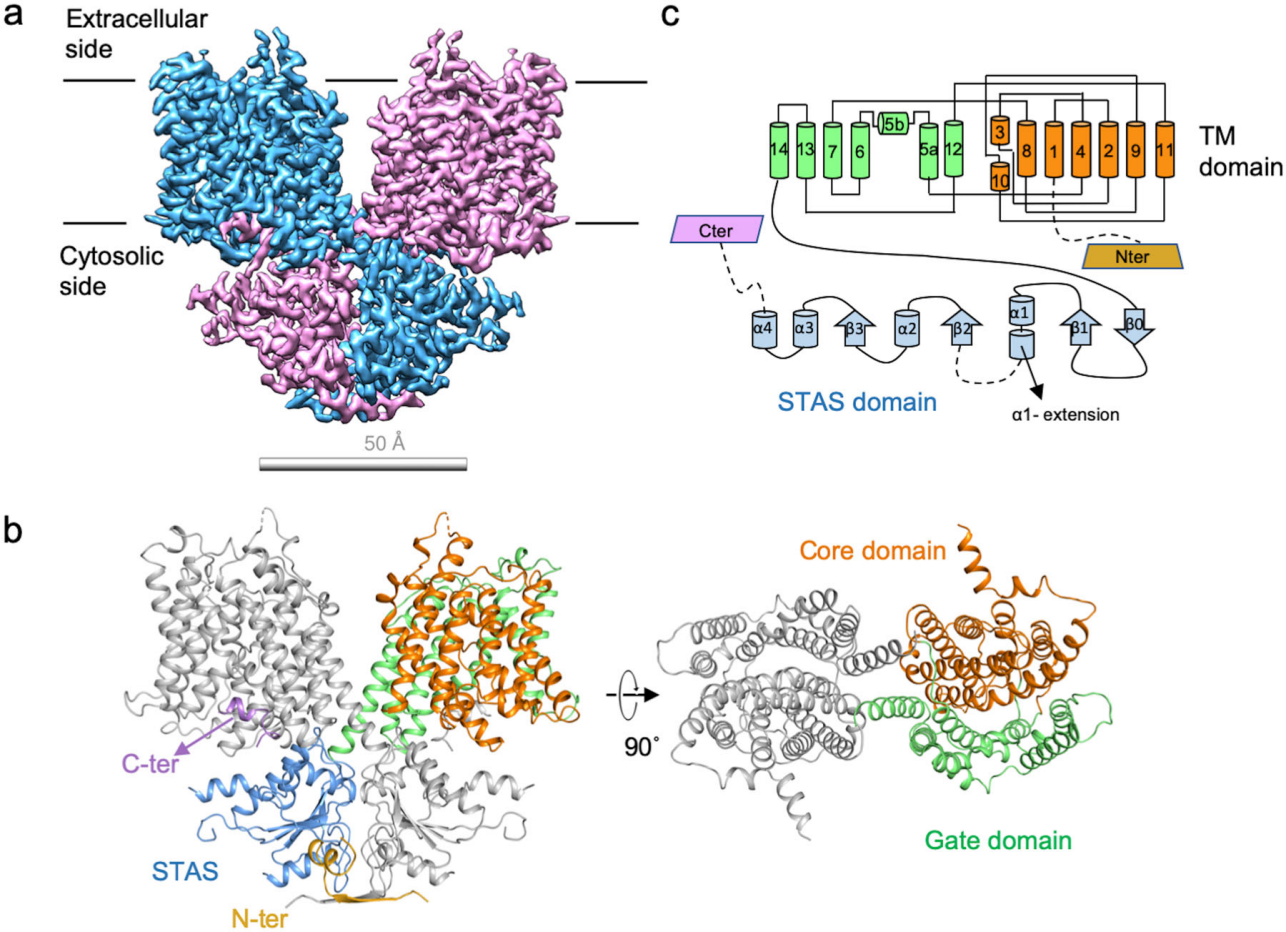
Structure of the human SLC26A9 homodimer. **a** Cryo-EM map and overall structure of the human SLC26A9 homodimer. Different subunits are colored in pink and blue. **b** Structure of the human SLC26A9 colored according to the different domains, with the N-terminus (N-ter) in gold, STAS domain in marine, C-terminus (C-ter) in violet, core domain in orange, and gate domain in lime. **c** Topology of human SLC26A9. The transmembrane (TM) segments are numbered from 1 to 14. The unwound region is formed by the two half-helices TM3 and TM10. An additional helix is located between TM6 and TM5 and is therefore labeled TM5b. The secondary structure of STAS domains is shown. All are colored accordingly.

To further understand the working mechanism for SLC26A9, we determined a cryo-EM structure of the full-length human SLC26A9 in glycol-diosgenin (GDN) at an overall resolution of 2.6 Å (Fig. 1a; Supplementary Figs. S1, S2 and Table S1), which allows unambiguous model building with the retention of robust sidechain information. All of the sequences are well resolved in the cryo-EM map except for several flexible regions, including the intervening sequence of the sulfate transporters and antisigma-factor antagonists domain, the region linking the STAS domain and the C-terminus, and the extracellular loop between TM3 and TM4. This structure exists as a homodimer, as previously reported for structurally related proteins, such as UraA^29^, SLC4^30,31^, and mouse Slc26a9^28^. The protomer can be divided into a TM domain and a large cytosolic STAS domain (Fig. 1b, c). The TM domain of SLC26A9 adopts a UraA-like “7 + 7” fold in which the TM1–TM7 segments are correlated with TM8–TM14 via a pseudo-C2 symmetry axis that is parallel to the membrane. The TM1–4 and TM8–11 form the core domain, which is surrounded by the gate domain formed by TM5–7 and TM12–14 (Fig. 1b, c). Both TM3 and TM10 exhibit a half-unwound helix packed with the remaining helix, together forming the canonical substrate-binding pocket in the middle, similar to UraA^29^ and UapA^32^. The rest of the TM segments in the core domain assemble in a horseshoe shape surrounding TM3 and TM10 in the center. TM segments of the gate domain stand along a thin line, with TM5 and TM12 facing the substrate-binding pocket flanked by TM13–TM14 and TM6–TM7 on both sides. The hydrophilic residues around the kink of TM12 not only provide structural flexibility but also interact with the neighboring TM5 through a salt bridge, enhancing the structural integrality of the gate domain. An additional TM5b helix is solved in human SLC26A9, which is a unique structural feature that has been previously reported in only mouse Slc26a9. TM5b supports the current position of TM13 and TM14, facilitating the proper assembly of the STAS domain (Supplementary Fig. S3a). The two protomers faced the sides of TM13 and TM14. A large gap is observed between the two TM domains of different protomers, wherein several patches of rod-like nonprotein density can be detected. The lack of shape features makes it difficult to conclude whether this density is a phospholipid or a GDN molecule, indicating that the binding is not specific. Along with the valine zipper (V zipper) between TM14 from different protomers, a large hydrophobic interface induces the dimerization of TM domains (Supplementary Fig. S3b).

The cross-shaped arrangement of the STAS domain and TM domain makes the two protomers intertwine together. The intracellular STAS domain is linked to TM14. A short helix after α1 in STAS (α1-extension), as part of the intervening STAS sequence, is actually well-folded and functionally connects the STAS domain to TM8 from another protomer through hydrophilic interactions between Q559 and N357, and a salt bridge between K560 and D362 further enhances the interdomain packing (Supplementary Fig. S3c). The N-terminal sequence of SLC26A9 is located in the bottom of the structure, forms a pair of antiparallel β-strand sheets, and partially sequesters the STAS domain from the cytosolic side. The hydrogen bond network (Supplementary Fig. S3d, scarlet frame) and hydrophobic interactions (Supplementary Fig. S3d, lime frame) formed by the N-terminal sequence together with the surrounding regions of the STAS domain contribute to SLC26A9 dimerization (Supplementary Fig. S3d). The C-terminal sequence, which contains a short amphipathic helix and a short loop, is bound to the cytosolic entrance of the putative ion-conducting pathway. The PDZ motif of SLC26A9 in the very end of the C-terminus was not built in our structure due to the lack of corresponding cryo-EM map densities.

### C-terminal sequence as a gating modulator

**Fig. 2:**
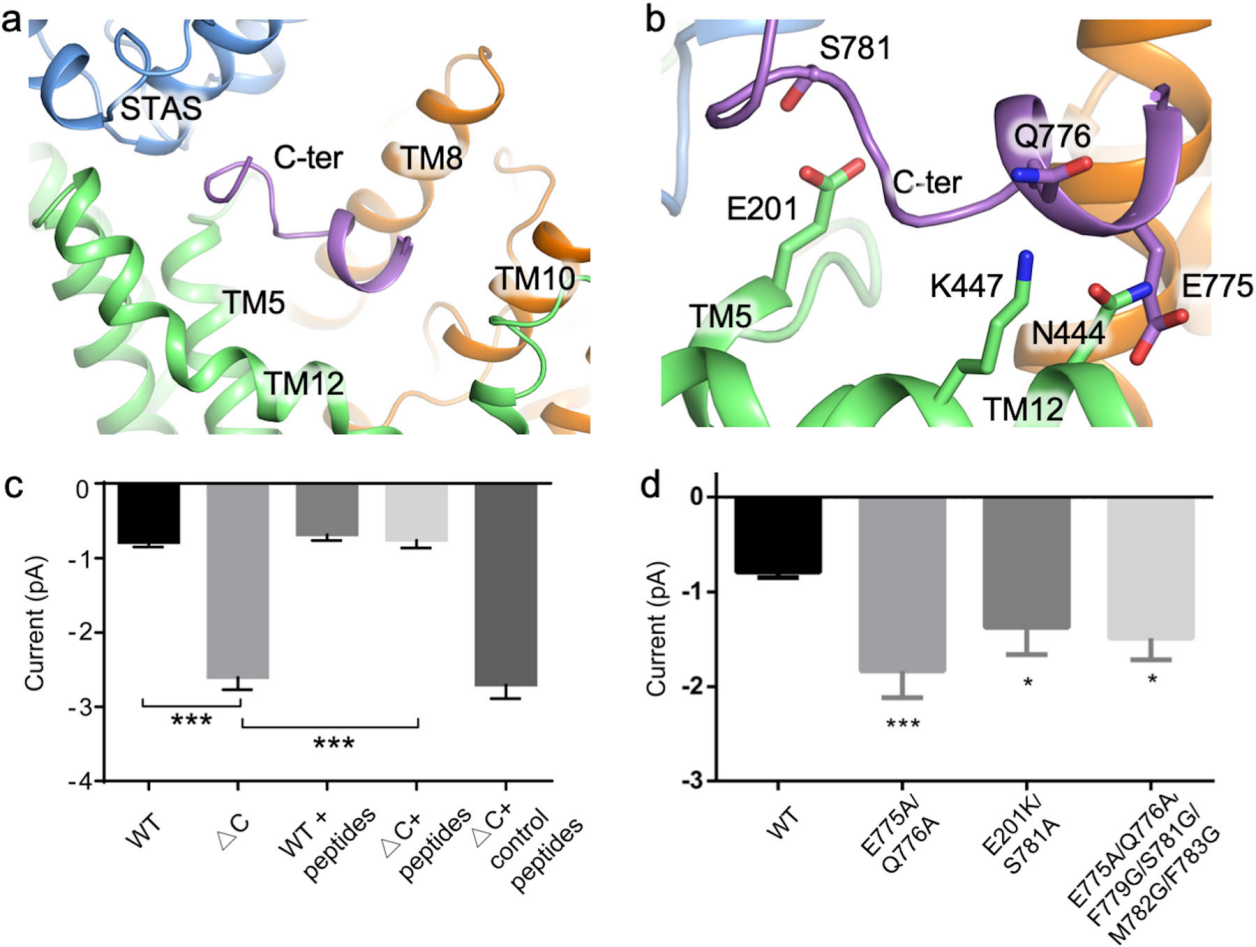
C-terminus binding alters transport function. **a** The C-terminal sequence is located in the cytosolic vestibule. **b** Hydrophilic interactions of the C-terminal sequence, which anchored the C-terminal sequence through interaction with TM5 and TM12. **c** Electrophysiological evidence for C-terminus gating, the cell numbers patched for each group were *n* = 19, 16, 11, 14, 11, respectively, ***P < 0.001. The sequence of the “peptides” is DLEQEMFGSMFH, which is the same as C-terminal sequence. The sequence of the “control peptide” is YEVHHQKLVFF. The same concentrations of both peptides were applied in the experiments. **d** Electrophysiology study of mutants that are involved in the C-terminal sequence binding. The currents recorded were –0.79 ± 0.06, –1.84 ± 0.28, –1.37 ± 0.29, –1.49 ± 0.23 pA and the cell numbers patched for each group were *n* = 19, 6, 8, 6, respectively, *P < 0.05, ***P < 0.001.

In our full-length structure, we observed an unexpected density located in the intracellular pocket lined by TM5, 8, 10, and 12. Further analysis shows that this density belongs to the C-terminal sequence (Fig. 2a). Sequence similarity analysis revealed poor conservation, indicating that the C-terminal sequence feature is unique to mammalian SLC26A9 (Supplementary Fig. S4). Because of the missing structure of the linkage sequence, we cannot determine which protomer the C-terminal sequence comes from.

This sequence folds into an amphipathic helix plus a hydrophobic loop that is bound to the TM segments through hydrophobic interactions. There are two patches of electrostatic interactions. One of them lies between the hydrophilic part of the helix involving Q776 and E775 and the kink point of TM12 (K447 and N444), and the other lies between S781 and E201 of TM5 (Fig. 2b).

Compared with the mouse Slc26a9 structure^28^, our structure exhibits no obvious conformational change (Supplementary Fig. S5a), indicating that the C-terminal sequence binding is unrelated to protein conformation regulation. However, an analysis of surface electrostatic potentials indicates that this binding alters the surface charge. The binding of the C-terminus buries most of the positively charged surface and places it with negative charge (Supplementary Fig. S5b). In addition, occupation by the C-terminus reduces the accessibility from the cytosolic side, placing an additional constriction site in the permeation pathway (Supplementary Fig. S5c). This may slow anion transport due to electrical repulsion and contraction of the ion permeation path. The binding mode of the C-terminal sequence observed here prompts its possible role in the modulation of channel opening.

To test the role of the C-terminus, we generated a construct that lacks the C-terminal sequence of SLC26A9 (residues 773–784) that is denoted by ΔC hereafter. However, heavy leakage in single-cell electrophysiological recordings of ΔC hampered the robustness of the conclusion. Therefore, single-channel recordings were applied to test the effect of the C-terminal sequence deletion. The results showed that the amplitude of ΔC single-channel currents was three times larger than that of wild-type SLC26A9, at –2.60 ± 0.16 and –0.79 ± 0.06 pA, respectively (Fig. 2c; Supplementary Fig. S5d). When synthesized peptides of the C-terminal sequence (0.5 mM) were added to the extracellular buffer, the ΔC construct was incompletely blocked and had similar amplitudes of the single-channel currents as the wild type: –0.77 ± 0.10 and –0.69 ± 0.08 pA, respectively (Fig. 2c; Supplementary Fig. S5d). To avoid possible structural interference, mutations of the C-terminal sequence were tested, including E775A/Q776A/F779G/S781G/M782G/F783G, E775A/Q776A, and E201K/S781A. Increased current was observed with the designed constructs, which means that the mutations could destroy the C-terminal sequence interaction with TM12 and TM5 simultaneously or separately (Fig. 2d). Hence the intact binding network of the C-terminal sequence is vital for inhibition.

### Ion coordination and transport properties

**Fig. 3:**
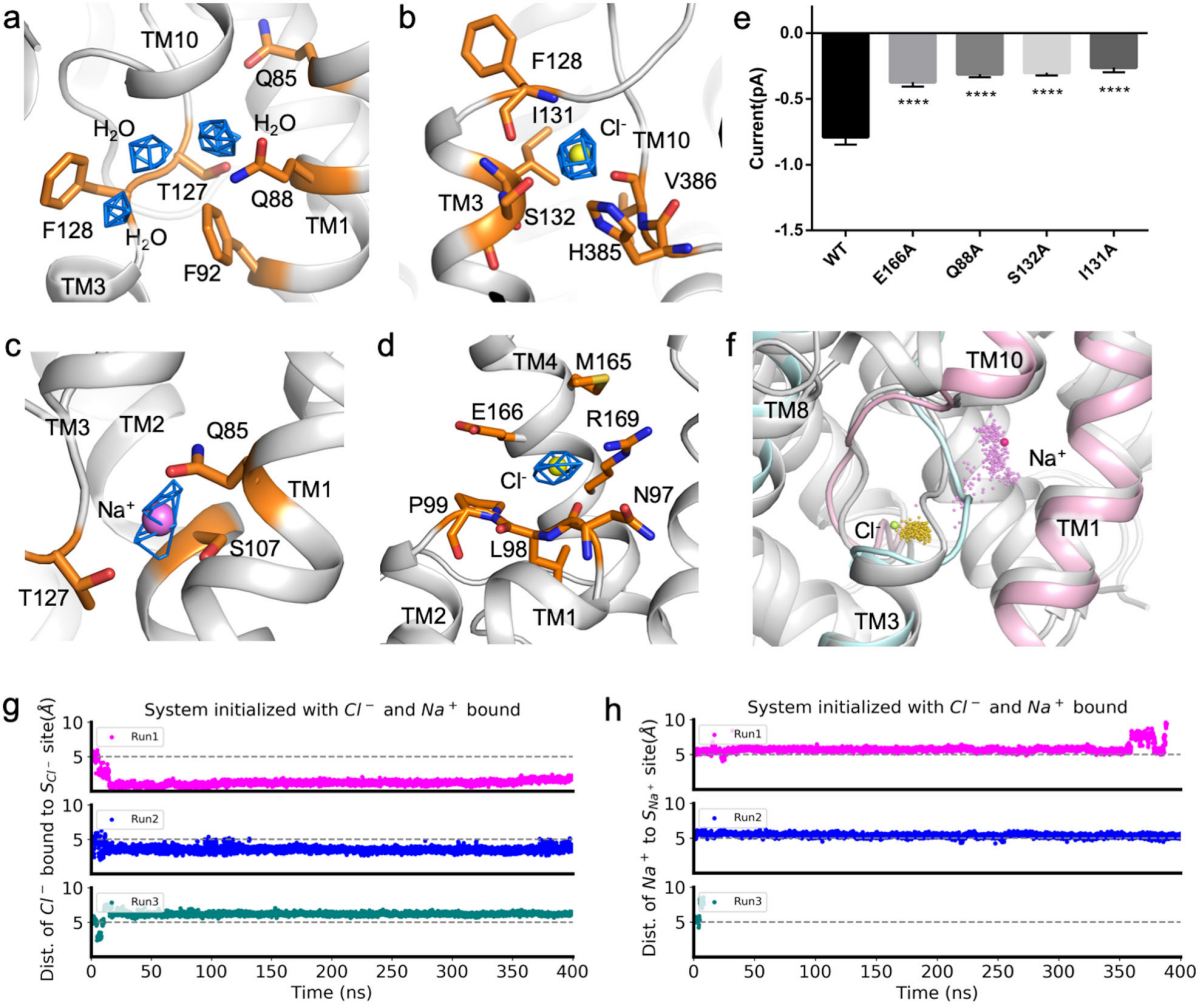
Ions and water molecules are resolved in human SLC26A9 structures. **a** Three water molecules are observed in the traditional substrate-binding pocket. Residues participating in coordination are labeled. **b** A chloride ion is identified near the N-terminus of the TM3 α-helix. **c** A sodium ion is coordinated through the hydroxyl groups of S107, T127, and Q85. **d** A chloride ion is bound near the extracellular side of SLC26A9. Residues from the TM1–TM2 loop and TM4 participate in coordination. **e** Electrophysiology of mutants that are involved in ion coordination. The currents recorded were –0.79 ± 0.06, –0.37 ± 0.03, –0.31 ± 0.02, –0.30 ± 0.02, and –0.26 ± 0.03 pA, and the cell numbers patched for each group were *n* = 19, 10, 10, 6, and 6, respectively, ****P < 0.0001. **f** Molecular dynamics simulation analysis of the sodium and chloride ion-binding sites. The TM10, TM1 and TM3, TM8 of the modeled structure are colored in pink and cyan, respectively. Modeled densities of Na^+^ and Cl^–^ are shown in pink and yellow dots, respectively. The positions of the ions in the cryo-EM structure are illustrated as magenta and green spheres. No apparent disagreement is observed between the modeled and actual positions of the ions. **g** The distance to site is plotted against time for Cl^–^ ions. **h** The distance to site is plotted against time for Na^+^ ions.

The high resolution of the solved SLC26A9 structure shows dozens of nonprotein densities in the cryo-EM map, among which two possible chloride ions and a sodium ion are built in the structure (Fig. 3a–d).

The traditional substrate-binding site between the half-helices TM3 and TM10 is occupied by a series of water molecules (Fig. 3a; Supplementary Fig. S6a). MD simulation indicates the unstable binding of Cl^–^ and Na^+^ to these sites, corresponding to the rapid Cl^–^ flux observed in SLC26A9, with Cl^–^ being the main ion transported^28^. As the traditional substrate-binding pocket can be accessed from the cytosolic side in the absence of the C-terminal sequence, the structure we captured is in the inward-open conformation. Mutations of the residues coordinating the water molecules influence Cl^–^ permeation (Fig. 3e).

A chloride ion is built in the cavity formed by TM3 and the loop half of TM10, coordinated by the sidechains of I131, S132, and H385. Carbonyl group from the main chain of F128 and V386 also facilitate ion binding (Fig. 3b). This ion-binding site is previously unreported, and is distinct from the canonical substrate-binding site. MD simulation revealed the stable binding of a chloride ion here (Fig. 3f, g). Mutation of either I131 or S132 decreases the ion current of human SLC26A9 (Fig. 3e).

An unreported sodium ion-binding site is identified in the center of the core domain, surrounded by TM1, TM2, and the loop half of TM3 and coordinated by Q85, T127, and S107 (Fig. 3c). A sodium ion is built here, as sodium ions are usually coordinated by hydroxyl groups, while Cl^–^ rarely lacks N coordination. This is further supported by MD results indicating that Na^+^ is the only ion that is stable here (Fig. 3f, h). The S107A mutant showed a significant difference from the wild-type strain in the whole-cell electrophysiology study when there was sodium or no sodium in the bath solution (Supplementary Fig. S6b). Furthermore, the residues participating in the coordination of Cl^–^ and Na^+^ in the central pocket are highly conserved (Supplementary Fig. S6c).

Another chloride ion is bound to a site near the extracellular vestibule and is coordinated by M165, E166, and R169 from TM4 and N97, L98, and P99 from the TM1–2 linker (Fig. 3d). This site is exposed to the extracellular solvent environment. MD simulation inferred the dynamic binding of chloride ions. The conformational change in the TM3–4 linker is predicted to coordinate chloride binding (Supplementary Fig. S6d, e). Mutations of E166 diminished ion flux, confirming that the ion binding is functional (Fig. 3e).

## Discussion

The structure of the full-length human SLC26A9 reported in this work uncovers SLC26A9 domain assembly and dimerization and, more importantly, reveals the function of the C-terminal sequence in ion permeation activity and ion coordination in the transport path. Many dimerization interfaces were observed, demonstrating that the human SLC26A9 likely exists as a dimer. A unique C-terminal sequence was bound in the entry of the intracellular vestibule of the protein, resulting in alternation of the surface electrostatics and blockage of the permeation path, thus slowing down ion permeation. Mutations in the C-terminal sequence hamper the inhibitory effect. Patches of nonprotein densities are observed around the intersection of the unwound half of TM3 and TM10. The traditional substrate-binding pocket of UraA or mouse Slc26a9 is occupied by several water molecules. Undefined pockets inside the core domain harboring sodium and chloride ions were revealed in our structure, which further enhanced the structural stability of the core domain. As the ions are sequestered from the ion permeation path, we prefer that the structural or regulatory coordination results in rather transient ion permeation. Interestingly, the binding of these two ions is dependent on the water molecules in the traditional substrate pocket. Since the transporter is captured in the inward-open formation, the water molecules and ion-binding pattern described here represent the most stable state. Mutations that affect ion or water coordination result in functional disruption. More research is needed to illustrate the mechanism of ion coordination during the ion permeation process.

**Fig. 4:**
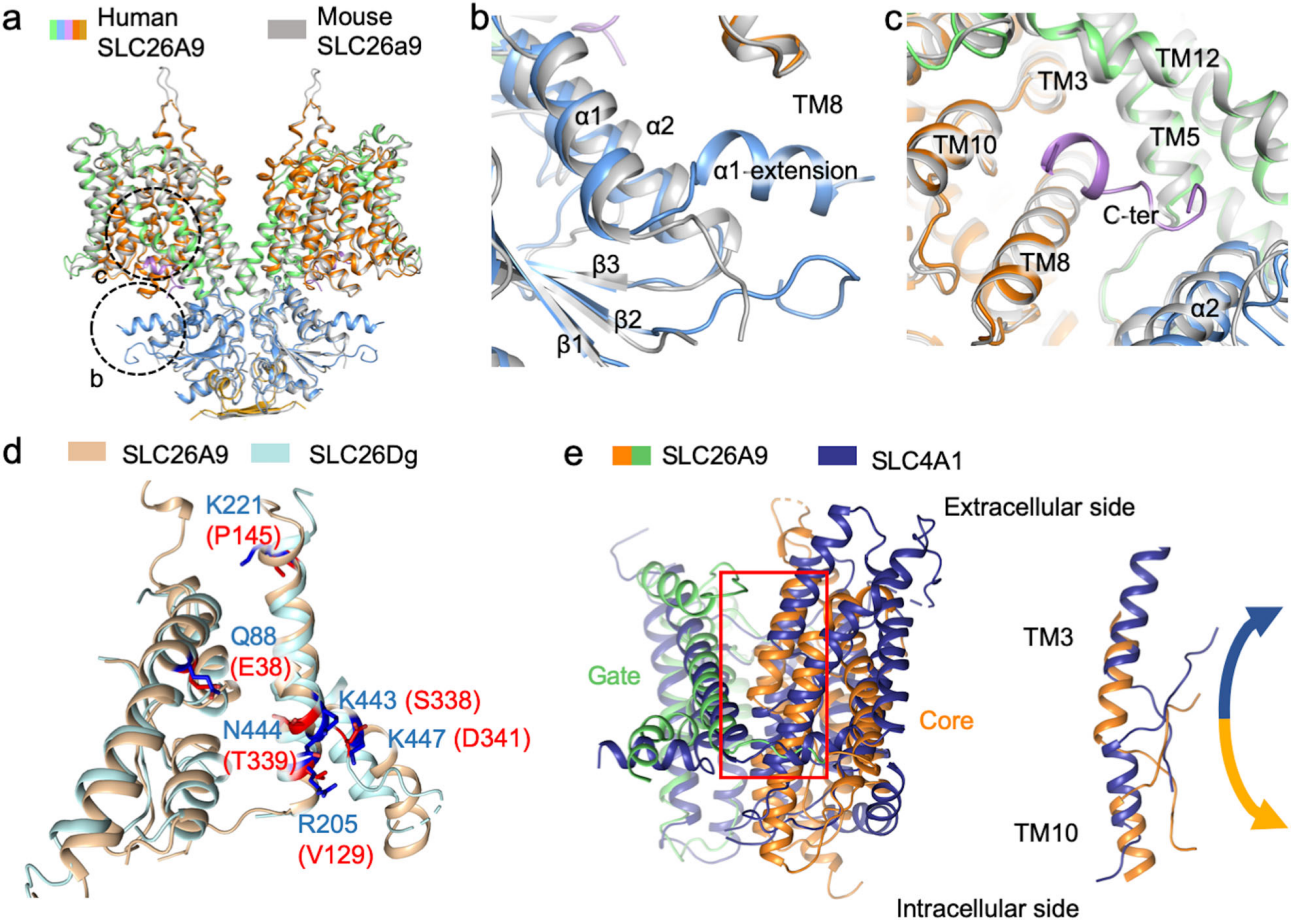
Comparison between the SLC26A9 structure and similar structures. **a** A superposition of the structures of human SLC26A9 and mouse Slc26a9. The major difference lies in the α extension of the STAS domain and the C-terminal sequence. **b** Enlarged view of the α extension. **c** Enlarged view of the C-terminal sequence. **d** Human SLC26A9 and SLC26Dg (PDB ID: 5IOF) aligned by the ion permeation pathway. SLC26Dg is colored cyan, and SLC26A9 is colored brown. The substituted residues are colored according to their relative charge, with blue representing more electropositivity and red representing more electronegativity. **e** Human SLC26A9 and human SLC4A1 (PDB ID: 4YZF) aligned by their gate domains. As SLC4A1 is solved in the outward-facing conformation, conformation differences in the core domains further support the elevator alternative-access model of SLC26A9. TM3 and TM10 are enlarged to depict the movement.

No obvious conformational change is observed in this structure compared with the mouse Slc26a9 structure in detergent. However, minor but important differences are located in the α1-extension and C-terminal sequence (Fig. 4a). The former directly links STAS and TM domains (Fig. 4b), while the latter, which is described in detail above, acts as an inhibitory element (Fig. 4c). While mouse Slc26a9 and human SLC26A9 exhibit several channel-like characteristics, SLC26Dg still behaves as a typical transporter^27^. There are several substitutions of residues along the transport path when they are compared with those of SLC26Dg (PDB ID: 5IOF), these substitutions increase the attraction of intracellular chloride ions (Fig. 4d). When SLC26A9 is aligned with the SLC4A1^33^ gate domain (PDB ID: 4YZF), significant rotation of the core domain can be detected, as it moves toward the extracellular side (Fig. 4e), and the flexible linker between the gate and core domains facilitates the movement. The substrate binding remains unchanged while core domain rocks relative to scaffold domain to gain accessibility to either side of the membrane. This further supports the elevator alternating-access mechanism^27,34^ of SLC26 family transporters, though they still exhibit channel-like activity^28^.

In conclusion, our work proposes a new gating mechanism for SLC26A9 mediated by the C-terminal sequence with potential pharmacological applications in treating dysfunction in respiration and digestion systems.

## Materials and methods

### Protein expression and purification

The full-length DNA segment for human SLC26A9 (UniProt ID: Q7LBE3, with K64N) was obtained by PCR from a cDNA library and subcloned into the pCAG vector^35^. The C-terminus-truncated construct (ΔC) was obtained by standard two-step PCR. The Flag tag was fused to the N-terminus of SLC26A9.

HEK293F (Invitrogen) cells were cultured in SMM T-1 medium (Sino Biological Inc.) under 5% CO_2_ and 37 °C provided by a Multitron-Pro shaker (INFORS, 130 rpm) Transfection was carried out when the cells reached a density of ~2.0 × 10^6^ cells/mL. Approximately 1.5 mg plasmids and 4 mg polyethylenimine (PEI, Polysciences, MW 25000) were applied to each liter of cells. After 48 h of culture, cells were harvested in lysis buffer containing 25 mM Tris, pH 8.0 and 150 mM NaCl by centrifugation at 800× *g* for 10 min. Then the cells were lysed with protein inhibitors (1 μg/mL pepstatin, 1.3 μg/mL aprotinin, 5 μg/mL leupeptin, and 0.2 mM phenylmethylsulfonyl fluoride (PMSF)) and detergents (1.5% n-dodecyl-β-D-maltopyranoside (DDM, Anatrace) and 0.3% cholesteryl hemisuccinate Tris salt (CHS, Anatrace)) at 4 °C for 2 h. After centrifugation at ~20,000× *g* for 1 h, the supernatant was applied to anti-Flag M2 affinity resin (Sigma) at 4 °C. The resin was washed with lysis buffer containing protein inhibitors and 0.02% glyco-diosgenin (GDN, Anatrace) and eluted in the same buffer with an additional 0.2 mg/mL Flag peptide. Then the protein was further purified by SEC (Superose 6 Increase 10/300 GL, GE Healthcare). The peak fraction was analyzed by SDS-PAGE and concentrated for cryo-EM sample preparation.

### Cryo-EM sample preparation and data acquisition

The purified SLC26A9 protein was concentrated to ~10 mg/mL. Aliquots (3 μL) of the protein were placed on glow-discharged holey carbon grids (Quantifoil Cu R1.2/1.3). The grids were blotted for 3.5 s and flash-frozen in liquid ethane cooled by liquid nitrogen with Vitrobot (Mark IV, Thermo Fisher Scientific). The prepared grids were transferred to a Titan Krios operating at 300 kV equipped with Cs corrector, Gatan K2 Summit detector and GIF Quantum energy filter. A total of 5281 movie stacks were automatically collected using AutoEMation^36^ with a slit width of 20 eV on the energy filter and a preset defocus range from –1.8 to –1.5 µm in super-resolution mode at a nominal magnification of ×105,000. Each stack was exposed for 5.6 s with an exposure time of 0.175 s/frame, resulting in a total of 32 frames/stack. The total dose rate was ~48 e^–^/Å^2^ for each stack. The stacks were motion corrected with MotionCor2^37^ and binned 2-fold, resulting in a pixel size of 1.091 Å. Meanwhile, dose weighting^38^ was performed. The defocus values were estimated with Gctf^39^.

### Data processing

A total of 2,137,248 particles were automatically picked using Relion 3^40–44^. After 2D classification, a total of 1,946,442 particles were selected. The selected particles were subjected to 3D classification by global angular searching against an initial model generated with Relion^45^ with C2 symmetry. For each of the last several iterations of this 3D classification, local angular searching was used to classify the particles into 4 classes. A total of 1,176,432 nonredundant particles were selected from the 3D classification using local angular searching. Then, these selected particles were subjected to multireference 3D classification and contrast transfer function (CTF) refinement^44^. The overall resolution of the 3D autorefinement structure after postprocessing was 2.6 Å. The final 3D reconstruction used 624,027 particles.

The 2D classification, 3D classification and autorefinement were performed with Relion 3. The resolution was estimated with the gold-standard Fourier shell correlation 0.143 criterion^46,47^ with high-resolution noise substitution^48^.

### Model building and structure refinement

Model building of SLC26A9 was performed ab initio with Coot^49^ based on the 2.6 Å cryo-EM maps with aromatic residues as positional markers, as most of these residues were clearly visible in our cryo-EM map. The position of each residue was manually checked using the chemical properties considered during model building.

A total of 638 amino acid residues were constructed for each monomer of SLC26A9. Several segments of the sequence were not modeled because the corresponding density was absent in the map.

Structure refinement was performed with Phenix^50^ with secondary structure and geometry restraints to prevent structure overfitting. To monitor the overfitting of the model, the model was refined against one of the two independent half maps from the gold-standard 3D refinement approach. Then, the refined model was tested against the other map^51^. Statistics associated with data collection, 3D reconstruction and model refinement can be found in Supplementary Table S1.

### Electrophysiology study of SLC26A9 and related constructs

HEK293T cells were maintained in standard cell culture conditions (37 °C, 5% CO_2_) in medium containing 90% Dulbecco’s modified Eagle’s medium (DMEM, Gibco) and 10% fetal bovine serum (FBS, Gibco). HEK293T cells were plated onto glass coverslips for subsequent patch-clamp recordings. After the confluency reached 30%–50%, the cells were transiently cotransfected using Lipofectamine 2000 (Invitrogen) with SLC26A9-, ΔC- or other mutants-expressing plasmid with an eGFP-encoding plasmid. Cells with green fluorescence were selected for patch-clamp recording 18–36 h after transfection.

All patch-clamp recordings were performed using an EPC10-USB amplifier (HEKA Elektronic), filtered at 3 kHz (low-pass Bessel filter) and sampled at 50 kHz. Data were acquired using Patchmaster software (HEKA Elektronic). All experiments were performed at room temperature. To avoid the influence of different protein expression levels between the wild-type and mutants on channel currents, we used single-channel recordings. Currents were obtained from excised membrane patches in the inside-out patch-clamp configuration. The borosilicate pipettes used had a resistance of 8–10 MΩ for single-channel recordings. Pipette solution contained: 145 mM N-methyl-D-glucamine (NMDG)-Cl, 1 mM MgCl_2_, 1 mM CaCl_2_, 10 mM HEPES, and 10 mM glucose (pH 7.4). The bath solution contained 145 mM NMDG-gluconate, 2 mM MgSO_4_, 10 mM HEPES, and 10 mM glucose (pH 7.4 with NMDG). Data were analyzed using Clampfit (Molecular Devices) after conversion by ABF Utility software. To calculate the amplitude of the channels, single-channel currents were recorded for 2–3 min at a holding potential of –100 mV. For the analysis, traces in which openings and closings were clearly observed were used to build all-point histograms after accounting for the leak current. The histograms for amplitudes were made by plotting against event numbers and the histograms were fitted to a Gaussian function where the peak corresponded with the unitary channel current amplitude. The relative area occupied by each Gaussian component therefore represents the relative frequency of events for each particular amplitude level.

For whole-cell patch-clamp recordings, currents were measured by a standard protocol that stepped the membrane potential from a 50-ms holding potential of 0 mV to 200-ms membrane potentials between –100 and +100 mV at 20 mV steps, before returning to 0 mV. The bath solution contained 145 mM NaCl, 1 mM MgCl_2_, 1 mM CaCl_2_, 10 mM HEPES, and 10 mM glucose (pH 7.4). The pipette solution contained 140 mM NaCl, 1 mM MgCl_2_, 10 mM HEPES, and 2 mM EGTA (pH 7.4). A Na^+^-free bath solution containing 145 mM NMDG-Cl, 1 mM MgCl_2_, 1 mM CaCl_2_, 10 mM HEPES, and 10 mM glucose (pH 7.4) was then perfused to examine the current changes. Current–voltage relationship curves were plotted subsequently. The borosilicate pipettes used had a resistance of 3–4.5 MΩ for whole-cell recordings. Data were analyzed using Origin (OriginLab) and GraphPad Prism (GraphPad Software). All data points are presented as the means ± standard error of the means (SEM), and *n* is the number of experimental cells from which recordings were obtained. Comparisons between two groups were made using an unpaired two-tailed *t*-tests as appropriate. Comparisons among three or more groups were made using one-way ANOVA analysis.

### Molecular dynamics simulations

The transmembrane domain of SLC26A9 is embedded into the lipid bilayers consisted of 233 POPC molecules, then solvated into the water box consisted of 20,340 TIP3P water molecules, with the 150 mM NaCl added to neutralize the system and maintain the ion concentration. The total number of the atom in the simulation system is about 99,700. All the MD simulations were carried out using CHARMM^52^ and OpenMM^53^, with molecules described by the CHARMM36m force field^54^.

The system was first minimized for 5000 steps and then equilibrated for 50 ps with 1 fs time-step and 500 ps with 2 fs time-step with gradual decrease of constraints on the heavy atoms. Finally, the extra 400 ns production simulations for analysis were performed with all atoms free under NPT ensemble with 2 fs time-step. The simulation temperature was set to 310 K, the pressure was maintained at 1 atm. Periodic boundary conditions, particle-mesh Ewald summation for electrostatic calculation, and the 12 Å switch-off distance for non-bond interaction calculation were used throughout the whole simulations. Monte Carlo membrane barostat was employed for the membrane stability with X–Y direction for isotropic and Z direction for free. The frames were saved every 100 ps and analyzed with VMD^55^ and MDTraj^56^ software packages.

We firstly built a set of simulation systems to identify the possible ions binding on each of nine potential ion-binding sites. As Cl^–^ or Na^+^ is far away from the majority of nine potential sites at the first 10 ns in MD simulation, we mainly established and analyzed the three simulation systems, including Cl^–^ bound to site 3, Na^+^ bound to site 8, and both Cl^–^/Na^+^ bound to their respective binding sites. Based on the ion remaining time and occupancy in the potential binding sites, the ion-binding stability of Cl^–^ and Na^+^ would be obtained.

## Supplementary information

Supplementary Information

## Data Availability

The atomic coordinates of human SLC26A9 have been deposited in the Protein Data Bank with the accession code 7CH1. The corresponding maps have been deposited in EMDB with the accession code EMD-30368.

## Supplementary information

**Supplementary Information** accompanies the paper at (10.1038/s41421-020-00193-7).
