# Supplementary material for: Structural insights into the gating mechanism of human SLC26A9 mediated by its C-terminal sequence
Source: Cell Discov. 2020 Aug 10;6:55. doi: 10.1038/s41421-020-00193-7 (PMC7417587; doi:10.1038/s41421-020-00193-7)
Supplement: Supplementary file 1 — Supplementary Information [file 41421_2020_193_MOESM1_ESM.pdf]

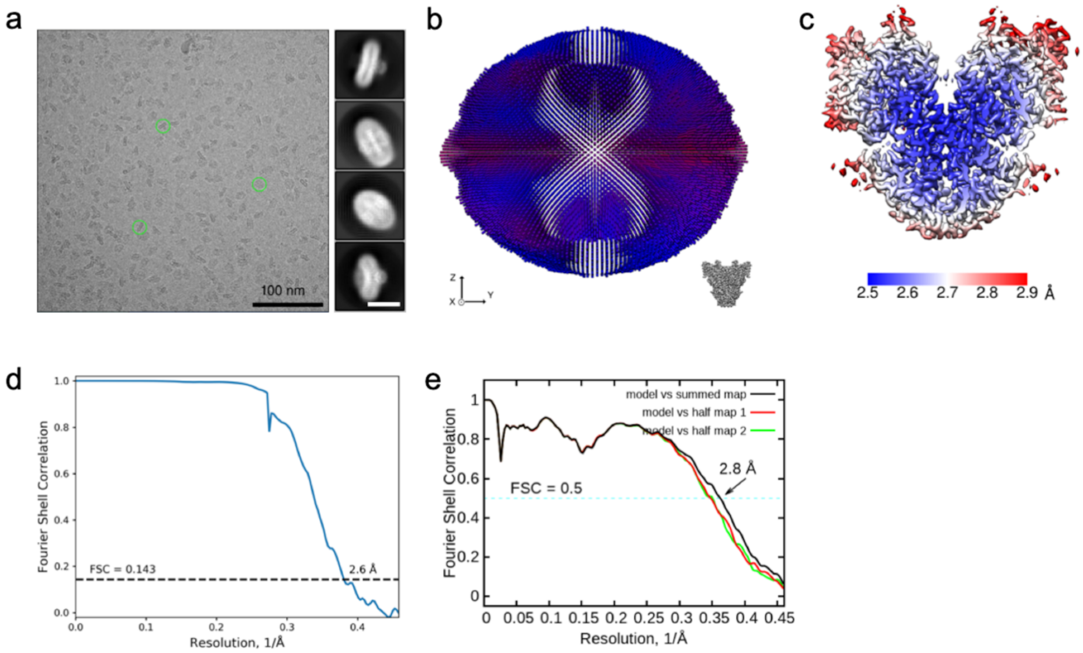

Supplementary Fig. S1 Cryo-EM analysis of SLC26A9. **a** Representative cryo-EM micrograph and 2D class averages. The scale bar in 2D class averages is 10 nm. **b-c** Euler angle distribution and Local resolution map for the 3D reconstruction of SLC26A9. **d** Gold standard FSC curves for the 3D refinement. **e** FSC curve of the refined model versus the overall cryo-EM map that it was refined against; of the model refined against the first half map versus the same map (red); and of the model refined against the first half map versus the second half map (green). The small difference between the red and green curves indicates that the refinement of the atomic coordinates did not suffer from overfitting.

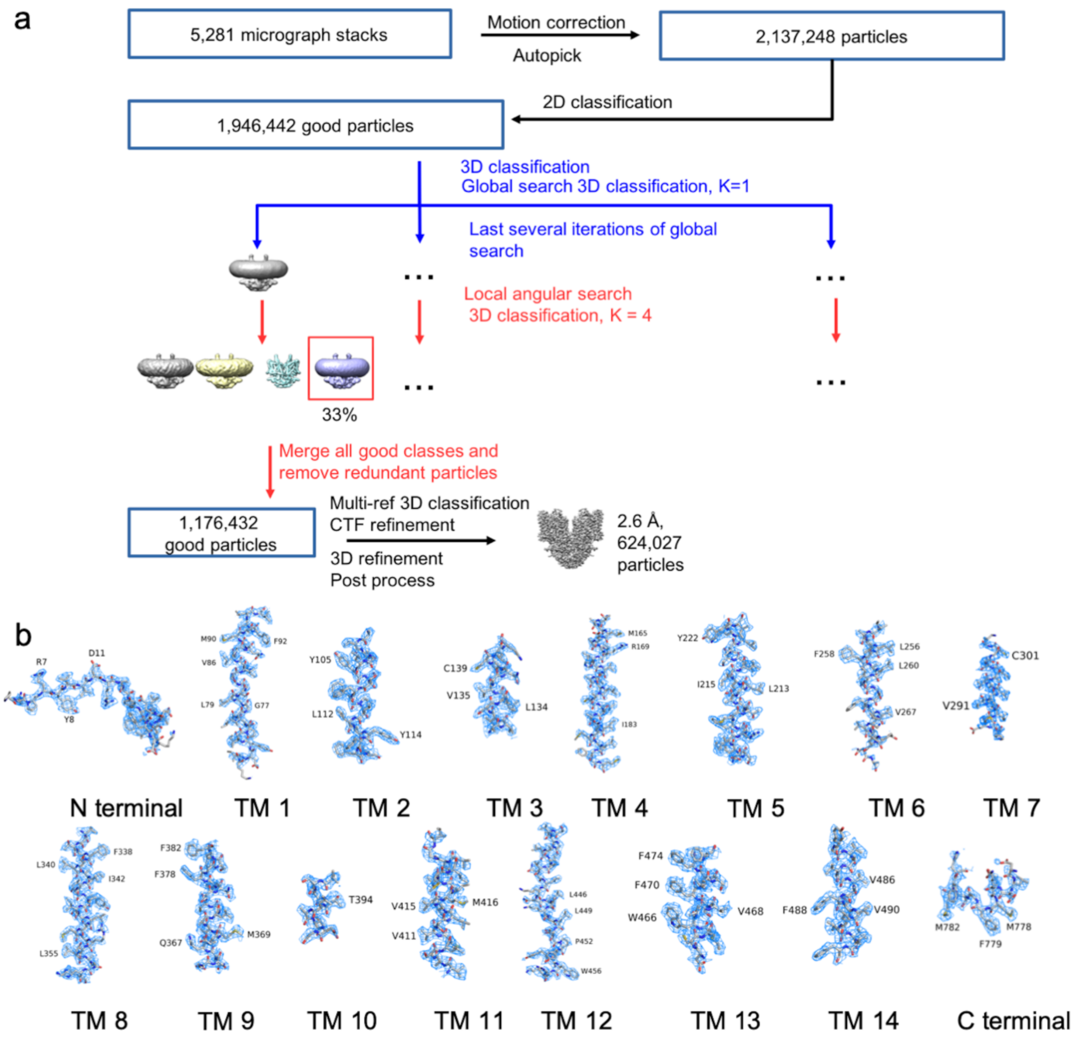

Supplementary Fig. S2 Cryo-EM data processing. **a** Flow-chart of Cryo-EM data processing. Please refer to the 'Data Processing' section in Materials and Methods for details. **b** Cryo EM density map of SLC26A9. The threshold is 8 σ.

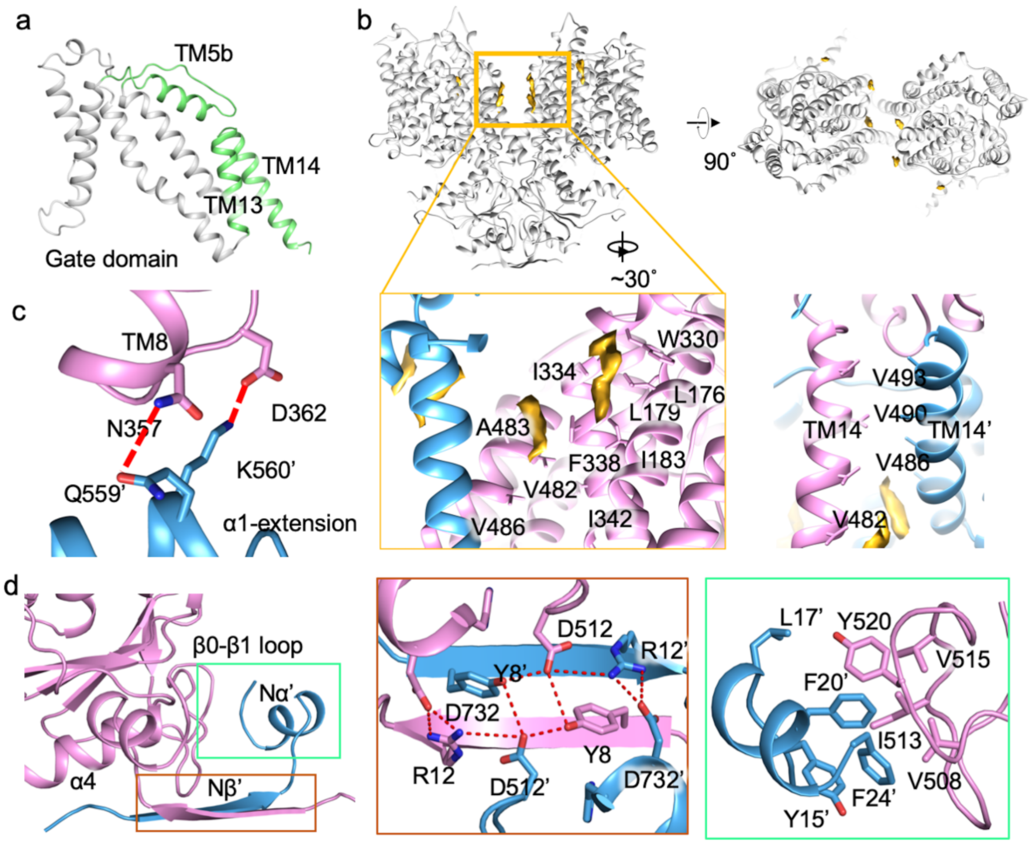

Supplementary Fig. S3 domain assembly of human SLC26A9. a TM5b is placed in the top of TM13 and TM14, which is absent in SLC4A1, UraA or SLC26Dg structures. **b** Lipid or detergent molecules are observed between the TM domains of the two protomers. Multiple hydrophobic residues participate in the binding. Top two insets: overview of the lipid or detergent molecules binding. Lipid or detergent molecules are shown in surface and colored gold. **c** Hydrophilic interaction between α-extension of STAS domain and TM8. **d** Dimerization induced through N-terminus. Mainly Nβ and Nα participate in dimerization. Scarlet insets: A hydrogen network formed by antiparallel Nβs from different subunits. H-bond are labeled by red dashed lines. Green insets: Hydrophobic interaction conducted by Nα’ and β0-β1 loop of the STAS domain.

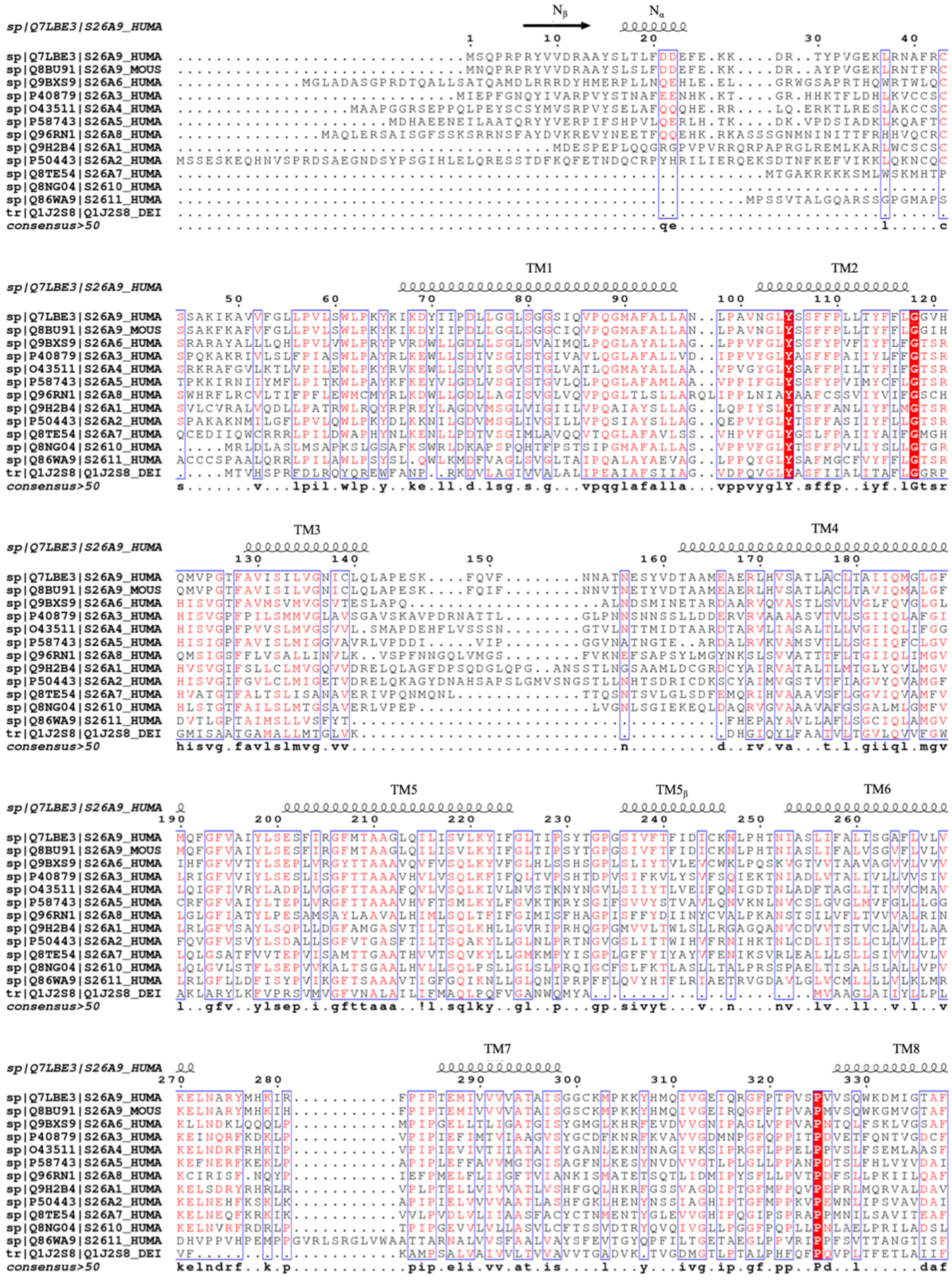

Nβ
Nα
TM1
TM2
TM3
TM4
TM5
TM5β
TM6
TM7
TM8
sp|Q7LBE3|S26A9_HUMA
sp|Q8BU91|S26A9_MOUS
sp|Q9BXS9|S26A6_HUMA
sp|P40879|S26A3_HUMA
sp|O43511|S26A4_HUMA
sp|P58743|S26A5_HUMA
sp|Q96RN1|S26A8_HUMA
sp|Q9H2B4|S26A1_HUMA
sp|P50443|S26A2_HUMA
sp|Q8TE54|S26A7_HUMA
sp|Q8NG04|S2610_HUMA
sp|Q86WA9|S2611_HUMA
tr|Q1J2S8|Q1J2S8_DEI
consensus>50

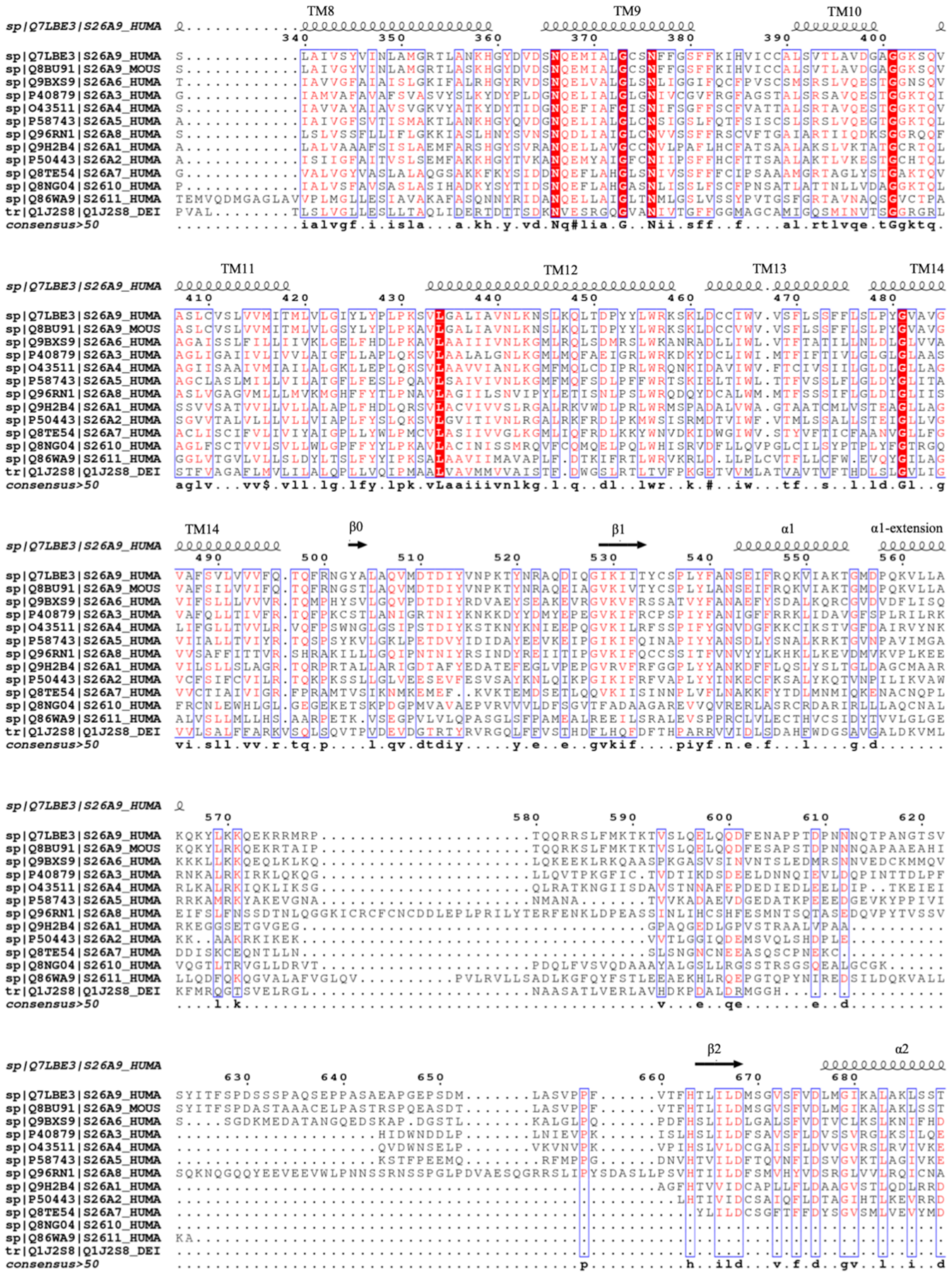

TM8
TM9
TM10
TM11
TM12
TM13
TM14
β0
β1
α1
α1-extension
β2
α2
sp|Q7LBE3|S26A9_HUMA
sp|Q8BU91|S26A9_MOUS
sp|Q9BXS9|S26A6_HUMA
sp|P40879|S26A3_HUMA
sp|O43511|S26A4_HUMA
sp|P58743|S26A5_HUMA
sp|Q96RN1|S26A8_HUMA
sp|Q9H2B4|S26A1_HUMA
sp|P50443|S26A2_HUMA
sp|Q8TE54|S26A7_HUMA
sp|Q8NG04|S2610_HUMA
sp|Q86WA9|S2611_HUMA
tr|Q1J2S8|Q1J2S8_DEI
consensus>50

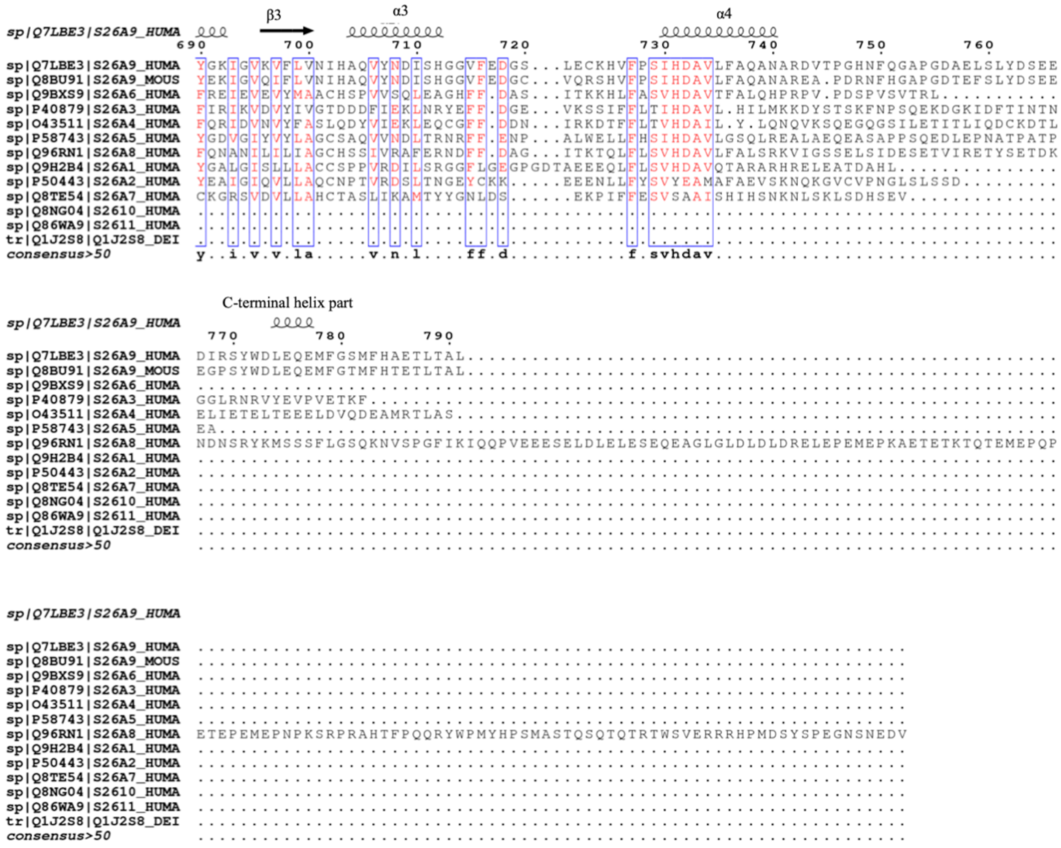

Supplementary Fig. S4 Sequence alignment of SLC26 homologues.

The sequence alignment of SLC26 homologues. The secondary elements are plotted in the top according to human SLC26A9 structure in this study. The sequences information can be accessed from UniprotKB[1]. The entry identifier is listed along with the protein name in the left.

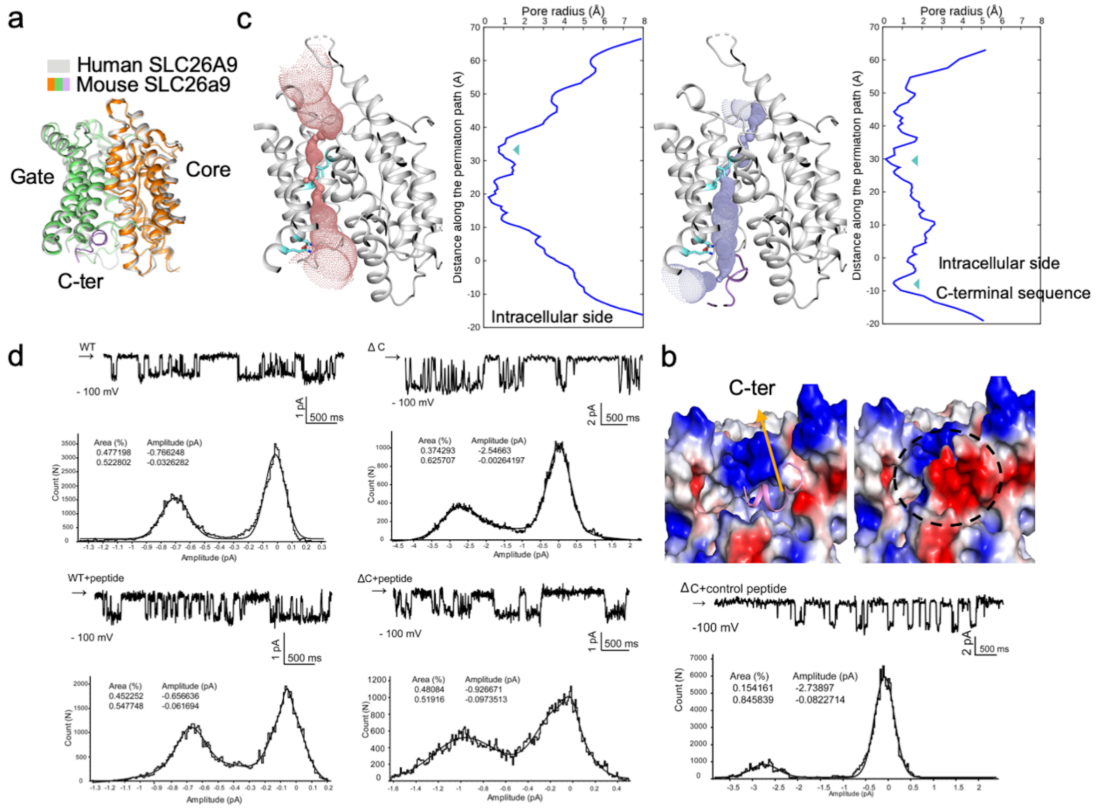

Supplementary Fig. S5 The function of C-terminal sequence of human SLC26A9.

a Superpose mouse Slc26a9 (PDB ID: 6RTC) with human SLC26A9 with RMSD 0.945 over 1196 Cα. Human SLC26A9 are colored by domains, while mouse Slc26a9 are colored in grey. The extension of 3α and C-terminus are highlighted by dashed circles. **b** Binding of the C terminus alters the electrostatic surface of the cytosolic entry for Cl^- ions, therefore may interfere the ion permeation. The electrostatic surface was generated by PyMOL[2]. **c** Cl^- permeation pathway calculated by HOLE[3] (brown spots). Two restriction sites can be detected in the Cl^- permeation path with key residues in TM domain and C terminus colored in aquamarine. Despite the residues inside the TM domain, an additional restriction site appears upon the C terminus binding. In the left is the pore radii along the permeation pathway. The width from extracellular to intracellular side of the protein were calculated. Arrowheads indicate

the approximate position of the restriction sites. **d** Single channel characterization of SLC26A9 and ΔC constructs. WT and ΔC recording without or with 0.5 mM peptide in the bath solution under -100 mV for 2-3 min.

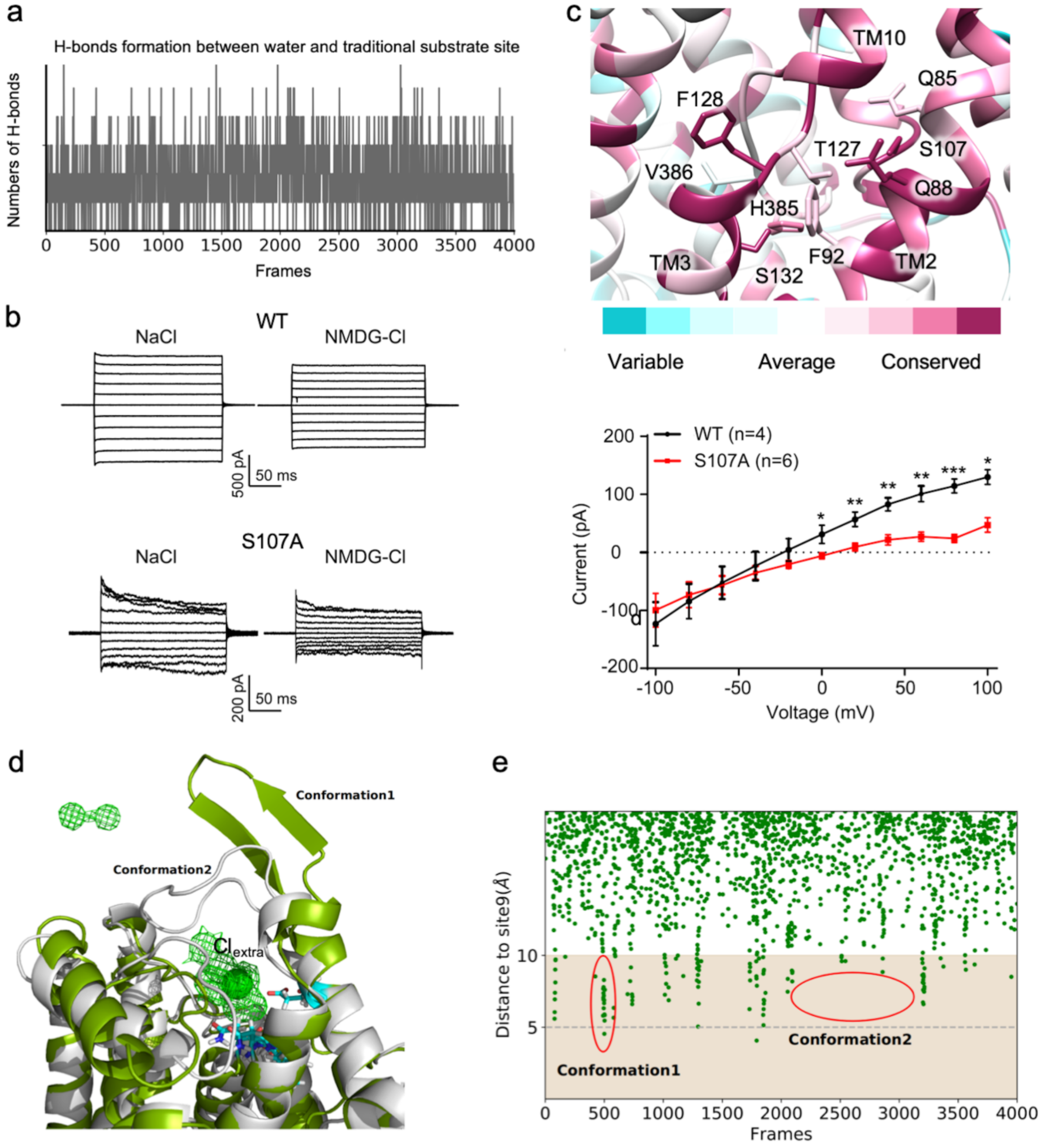

Supplementary Fig. S6 Ions and water molecules bound to SLC26A9. a Hydrogen bonds observed between the modeled water molecules and the traditional substrate binding sites. **b** Electrophysiology study of S107A with different bath solution. *P < 0.05, **P < 0.01, ***P <0.001.**c** The residues involved in the ions and water binding around the traditional substrate binding site are highly conserved. V386 is an exception probably due to its main-chain hydroxyl group participating in ion coordination. **d** The coordination of chloride ion near the extracellular vestibule is dependent on the conformation of TM3-TM4 loop. Cl_{extra} is available only in the conformation1. **e** the distance to site of the ions in **d** is plotted against frames.

Table S1 | Data collection, 3D reconstruction and model statistic

**Data collection**	
EM equipment	Titan Krios (Thermo Fisher Scientific)
Voltage (kV)	300
Detector	Gatan K2 Summit
Energy filter	Gatan GIF Quantum, 20 eV slit
Pixel size (Å)	1.091
Electron dose ($e^-/Å^2$)	48
Defocus range (μm)	-1.2 ~ -2.2
Number of collected micrographs	5,281
**3D Reconstruction**	
Software	Relion 3.0
Symmetry	C2
Number of used particles	624,027
Resolution (Å)	2.6
Map sharpening B-factor ($Å^2$)	-150
**Refinement**	
Software	Phenix
Cell dimensions	
a=b=c (Å)	349.12
α=β=γ (˚)	90
Model composition	
Protein residues	1,276
Side chains assigned	1,276
R.m.s deviations	
Bonds length (Å)	0.005
Bonds Angle (˚)	0.894
Ramachandran plot statistics (%)	
Preferred	94.75
Allowed	5.10
Outlier	0.16

References

1 UniProt, C. UniProt: a worldwide hub of protein knowledge. *Nucleic Acids Res* **47**, D506-D515, doi:10.1093/nar/gky1049 (2019).

2 Alexander, N., Woetzel, N. & Meiler, J. bcl::Cluster : A method for clustering biological molecules coupled with visualization in the Pymol Molecular Graphics System. *IEEE Int Conf Comput Adv Bio Med Sci* **2011**, 13-18, doi:10.1109/ICCABS.2011.5729867 (2011).

3 Smart, O. S., Neduvelil, J. G., Wang, X., Wallace, B. A. & Sansom, M. S. HOLE: a program for the analysis of the pore dimensions of ion channel structural models. *J Mol Graph* **14**, 354-360, 376 (1996).
